# Long-term PM_2.5_ exposure and the clinical application of machine learning for predicting incident atrial fibrillation

**DOI:** 10.1038/s41598-020-73537-8

**Published:** 2020-10-01

**Authors:** In-Soo Kim, Pil-Sung Yang, Eunsun Jang, Hyunjean Jung, Seng Chan You, Hee Tae Yu, Tae-Hoon Kim, Jae-Sun Uhm, Hui-Nam Pak, Moon-Hyoung Lee, Jong-Youn Kim, Boyoung Joung

**Affiliations:** 1 Division of Cardiology, Department of Internal Medicine, Severance Cardiovascular Hospital, Yonsei University College of Medicine, 50-1 Yonsei-ro, Seodaemun-gu, Seoul, 03722 Republic of Korea; 2 Division of Cardiology, Department of Internal Medicine, Gangnam Severance Hospital, Yonsei University College of Medicine, 211 Eonju-ro, Gangnam-gu, Seoul, 06273 Republic of Korea; 3 Department of Cardiology, CHA Bundang Medical Center, CHA University, Seongnam, Republic of Korea; 4 Department of Biomedical Informatics, Ajou University School of Medicine, Suwon-si, Gyeonggi-do Republic of Korea

**Keywords:** Cardiovascular diseases, Environmental impact

## Abstract

Clinical impact of fine particulate matter (PM_2.5_) air pollution on incident atrial fibrillation (AF) had not been well studied. We used integrated machine learning (ML) to build several incident AF prediction models that include average hourly measurements of PM_2.5_ for the 432,587 subjects of Korean general population. We compared these incident AF prediction models using c-index, net reclassification improvement index (NRI), and integrated discrimination improvement index (IDI). ML using the boosted ensemble method exhibited a higher c-index (0.845 [0.837–0.853]) than existing traditional regression models using CHA_2_DS_2_-VASc (0.654 [0.646–0.661]), CHADS_2_ (0.652 [0.646–0.657]), or HATCH (0.669 [0.661–0.676]) scores (each p < 0.001) for predicting incident AF. As feature selection algorithms identified PM_2.5_ as a highly important variable, we applied PM_2.5_ for predicting incident AF and constructed scoring systems. The prediction performances significantly increased compared with models without PM_2.5_ (c-indices: boosted ensemble ML, 0.954 [0.949–0.959]; PM-CHA_2_DS_2_-VASc, 0.859 [0.848–0.870]; PM-CHADS_2_, 0.823 [0.810–0.836]; or PM-HATCH score, 0.849 [0.837–0.860]; each interaction, p < 0.001; NRI and IDI were also positive). ML combining readily available clinical variables and PM_2.5_ data was found to predict incident AF better than models without PM_2.5_ or even established risk prediction approaches in the general population exposed to high air pollution levels.

## Introduction

Although atrial fibrillation (AF) often leads to complications such as stroke in patients without an awareness of such preexisting diseases, electrocardiogram screening is not sufficient to detect AF in the general population^1,2^. Some scoring systems for predicting incident AF have been introduced, including the CHADS_2_^3^, CHA_2_DS_2_-VASc^4^, and HATCH^5^ scores; however, their prediction accuracies are not sufficient for wide application. Although epidemiological studies have suggested that an elevated level of ambient particulate matter < 2.5 μm in aerodynamic diameter (PM_2.5_) is consistently associated with adverse cardiac events^6^ and arrhythmias^7^, including AF^8^, the role of PM_2.5_ on incident AF remains to be investigated. Recently, data-driven analyses using machine learning (ML) methods have been introduced to identify some blood biomarkers that are risk factors of AF prevalence (not incidence)^9^, and they were considered non-inferior to traditional analyses^9,10^. However, it was not clear whether these data-driven approaches could find correlations between PM_2.5_ and incident AF, or if they could predict incident AF better than traditional analysis in clinical practice.

Although some studies from Western countries did not show a correlation between short-term exposure to PM_2.5_ and incident AF^11,12^, the air pollution levels in those areas were much lower than the levels in Asian countries; therefore, the effect sizes could be low in those studies. Our previous study, performed in the general population of an Asian country, showed correlations between PM_2.5_ exposure and increased AF incidence^8^. Thus far, the identification of AF risk factors had been hypothesis driven and most studies performed analyses based on the selection of several cardiovascular risk factors. To perform a data-driven analysis for revealing AF risk factors, we used 27 readily available parameters including PM_2.5_ level in the Korean general population. All subjects without a history of previous AF were included in our population to identify the risk factors for incident AF. We also analyzed already revealed clinical risk factors to determine which risk factors best predict incident AF in this population. We investigated the robust risk factors for incident AF by using both the traditional regression method and the ML algorithm.

## Methods

In this nationwide cohort study, we investigated the relationship between long-term exposure to PM_2.5_ and incident AF by using ML methods. The study protocol adhered to the ethical guidelines of the 1975 Declaration of Helsinki. The protocol was approved by the Institutional Review Board of Yonsei University College of Medicine, which waived the need for informed consent.

### Data source

The National Health Insurance Service (NHIS) of Korea is a single-payer program that is mandatory for all residents of South Korea. Among the entire South Korean population (about 47 million people in 2002), 96.6% are registered in the NHIS. About 70% of the entire cohort had undergone a national health examination. This retrospective study used the NHIS-based National Sample Cohort (NSC) 2002–2013 dataset, comprising a random sample of 1,025,340 subjects, which amounted to 2.2% of the entire Korean population under the NHIS in 2002 (46,605,433). This sample cohort was extracted through probability sampling from all beneficiaries of the NHIS and the National Medical Aid in 2002 based on the entirety of the national cohort data. Systematic sampling was performed in each of the 1476 strata based on age, sex, eligibility status, socioeconomic status, and income level, with the sample size proportionate to the cohort size of the strata. The representativeness of the sample has been previously examined by comparing the sample to the entire Korean population^13^.

### Study cohort

**Figure 1:**
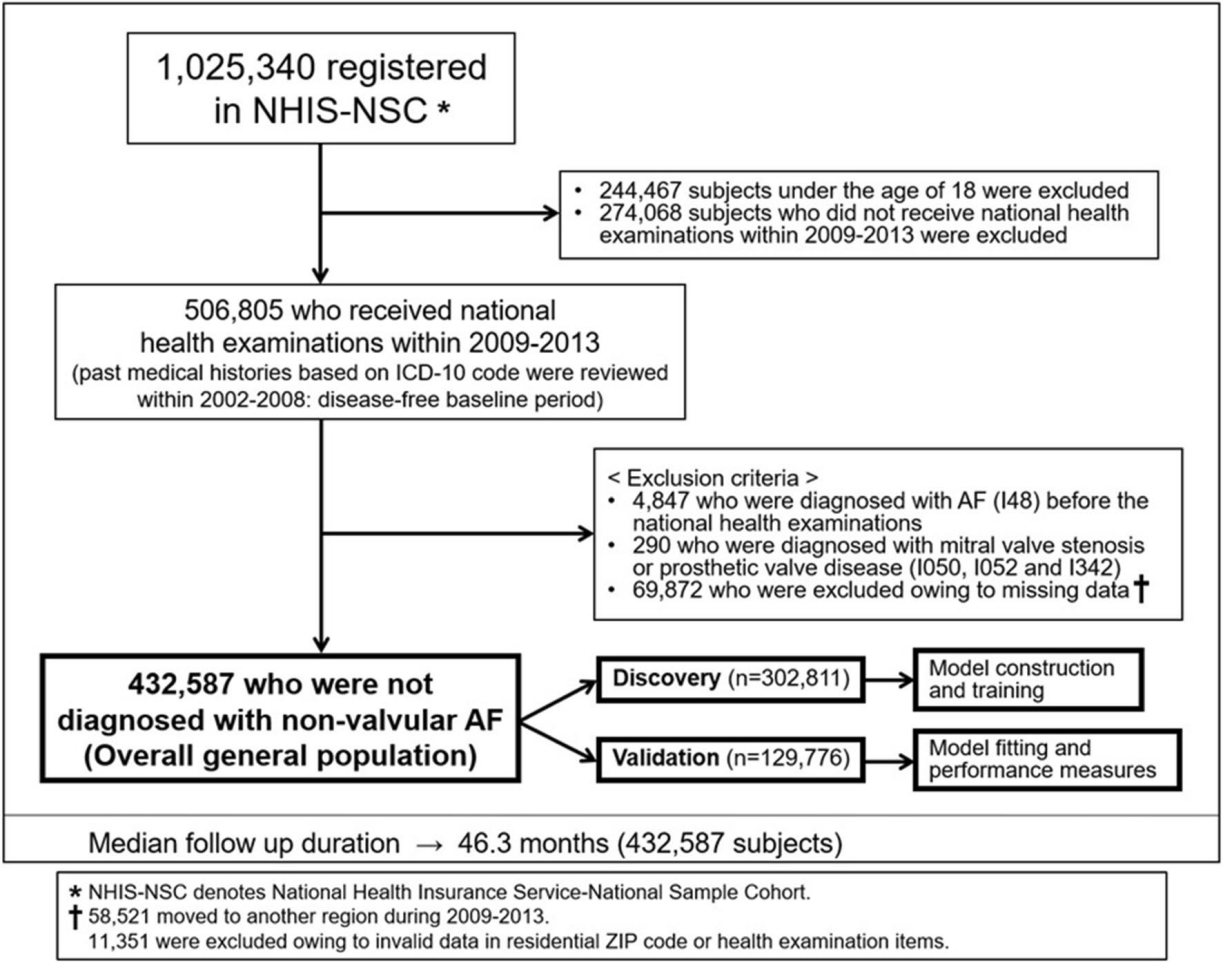
Study cohort and included subjects from the National Health Insurance Service National Sample Cohort (NHIS-NSC; overall general population). We randomly divided the population into the discovery (n = 302,811) and validation (n = 129,776) cohorts. In the discovery cohort, model construction and training were performed. Model fitting and performance measures were performed in the validation cohort. *AF* atrial fibrillation.

From the NHIS-NSC, adults (n = 506,805) older than 18 years who had undergone a national health examination at least once between 2009 and 2013 were selected^13^. Each subject was followed from the day of the health examination to December 31, 2013, and screened from January 2002 to assess the medical history (2002–2008: disease-free baseline period). Each diagnosis, based on the 10th revision of the International Classification of Disease codes, was defined as the first occurrence during at least two different days of outpatient hospital visits or on the first hospital admission (Supplementary Table 1)^14,15^. The following exclusion criteria were applied (Fig. 1): (i) age < 18 years; (ii) a diagnosis of AF before undergoing a health examination; (iii) valvular AF, such as mitral valve stenosis or prosthetic valve disease; (iv) change of residence to another ZIP code in 2009–2013; and (v) missing data on residential ZIP code or in questionnaires for smoking status and alcohol intake completed during the health examinations. A final population of 432,587 subjects was included in the analysis (Fig. 1). For the purpose of analysis, the subjects were divided chronologically in an approximately 7:3 ratio for a conventional discovery-validation approach.

### Air pollution measurements

During the study period, PM_2.5_ levels, temperature, and humidity were measured hourly at the 313 sites of the Korean Nationwide Meteorological Observatory by the Korean Department of Environmental Protection. The entire Korean peninsula is divided into 256 residential ZIP codes including 74 metropolitan areas (average 73 km^2^). To assess long-term PM_2.5_ exposure effects, the nearest monitoring facility of each residence was identified and used to assess the average annual pollutant levels for each study subject^16^. The geographically based long-term average PM_2.5_ level during the study period was measured hourly by the monitoring facilities^16^, and 256 residential ZIP codes were matched with the nearest monitoring facilities. Meteorological variables (temperature and humidity) were included as geographically based long-term averages of the hourly measured temperature and humidity for each subject during the follow-up period. The long-term average (during the total study period for each subject) PM_2.5_ levels and meteorological measurements (temperature and humidity) were calculated from these hourly measurements at each site. The Korean National Ambient Air Quality Standards (NAAQS) and PM_2.5_ measurement methods are described in Supplementary Table 2.

### Primary outcome

The primary outcome was the incidence of AF according to the PM_2.5_ level. AF was diagnosed on the basis of hospital admission or at least two outpatient visits for AF^17,18^. The cohort was followed up to the time of an AF incident, the time of disqualification from the NHIS (death or immigration), or the end of the study (December 31, 2013).

### Machine learning

Twenty-six readily available clinical parameters and PM_2.5_ data were used for variable selection. Several supervised ML classifiers included support vector machine (SVM), decision tree, random forest, Naïve Bayes, deep neural network, and extreme gradient boosting models. Our SVM model was used to differentiate patients whether they developed new-onset AF or not by a computed hyper-plane (the optimal cost and gamma parameters were found with radial kernel) which separates these categories most effectively^19^. Our decision tree model constructed with a recursive tree structure using computationally selected parameters can differentiate features step by step by creating appropriate splits (recursive partitioning). And it was combined by ensembled algorithms to construct better prediction models such as gradient boosting (500 iterations using root mean square error as evaluating metric) and random forest (ten decision trees were combined to construct the best model)^20^. As mathematical models, artificial neural network systems mimic human neural networks which can be trained to discriminate different patterns of diseases, and we selected the three-layered deep neural network model using Tensorflow backend with Keras framework^21^. The entire ML process consisted of ML with automated feature selection by information gain attributive ranking algorithm^22^, model constructing with a boosted ensemble algorithm, and tenfold cross-validation to reduce overfitting^23^.

On the basis of supervised ML methods to construct prediction models, we used a sequential method of feature construction and automated selection by information gain ranking to identify predictive risk factors from the various health examination parameters^22^. Variable selection with an entry criterion of p < 0.05 was applied, and data-driven approaches were used to identify the smallest number of variables required for each prediction model. By using each model, selected variables including PM_2.5_ were modeled for their association with incident AF in the discovery cohort, and subsequently evaluated in the validation cohort (Fig. 1). New-onset AF events were analyzed with the geographically based long-term average PM_2.5_ level during the study period for each subject. Bootstrapping and tenfold cross-validation were used to adjust the model coefficient to avoid overfitting in the discovery sample. Model accuracy was calculated in the validation sample (30% of the original dataset), and loss was calculated using binary or categorical cross-entropy. The area under the receiver operating characteristic curve or c-index was used for each constructed model, and the net reclassification improvement index (NRI) and integrated discrimination improvement index (IDI) were calculated to assess the additional discriminative ability of these models.

Networks for training and validation were constructed based on Tensorflow (version 1.10) using the Keras framework (version 2.1.6), and all statistical analyses were performed using R (version 3.5) and Python (version 3.6) software. Training and validation were performed on an Intel central processing unit Xeon Scalable Gold 6126 under two graphics processing unit (GPU) devices support (two Nvidia RTX 2080Ti GPU devices; CUDA version 9.0), and constructed models were saved for further analysis.

### Statistical analyses

The baseline characteristics of subjects with and without AF in both the discovery and validation cohorts were compared. We assumed that the study subjects were exposed to ambient air pollution within their residential ZIP codes during the study period^16^. Individual subjects were matched with the average air pollution levels and meteorological information during the study period obtained from the nearest monitoring facilities (according to the subjects’ residential address). By using Cox proportional-hazard model regression analysis, the relationship between incident AF and PM_2.5_ level was analyzed using a generalized estimating equation approach with a random-effect analysis^24^. The proportionality of the hazard assumptions was checked with a log-minus-log graph and a test on the Schoenfeld residuals. Consequently, the test results were found to be valid for each lifestyle factor. In Cox regression analysis, the included subjects were followed from their national health examination until the development of new-onset AF, disqualification (death or immigration), or the end of the study. A two-tailed p-value of < 0.05 was considered statistically significant.

## Results

### Baseline characteristics

There were no significant differences in body mass index (BMI), smoking history, socioeconomic status, and follow-up duration between the groups (Table 1). Subjects who developed new-onset AF during the follow-up were older, included a higher proportion of men, were more likely to have comorbidities, and were exposed to higher average PM_2.5_ levels than those without AF (Table 1). Subjects with AF had higher use of antiplatelet agents, beta-blockers, and statins than those without AF.

**Table 1:** Baseline characteristics of the study population (n = 432,587).

Variables	Discovery		Validation	
	No AF (n = 300,367)	Incident AF (n = 2,444)	No AF (n = 126,395)	Incident AF (n = 3,381)
**Age, years**	48.3 ± 14.1	63.1 ± 13.4	45.7 ± 14.5	62.8 ± 12.4
65–74 (%)	(11.3)	(30.3)	(7.5)	(35.2)
≥ 75 (%)	(3.2)	(19.6)	(3.4)	(15.0)
Male (%)	(50.5)	(53.2)	(46.9)	(57.6)
**BMI, kg/m**^**2**^	23.7 ± 3.2	24.2 ± 3.4	23.6 ± 3.5	24.3 ± 3.4
Obesity (BMI ≥ 27.5 kg/m^2^) (%)	(11.8)	(15.6)	(12.8)	(16.1)
**Smoking, pack·years**	6.1 ± 11.8	8.1 ± 15.3	5.8 ± 11.4	8.8 ± 15.8
Non- (%)	(62.6)	(63.4)	(62.0)	(61.8)
< 20 pyrs (%)	(24.3)	(17.6)	(25.5)	(17.6)
≥ 20 pyrs (%)	(13.1)	(19.0)	(12.5)	(20.6)
**Alcohol intake, g/week (mean)**	61.2 ± 13.7	45.9 ± 12.7	63.9 ± 14.4	55.8 ± 15.1
≥ 220.5 g/week	(7.8)	(6.3)	(8.3)	(7.7)
Socioeconomic status, higher* **(%)**	(60.9)	(62.6)	(59.3)	(62.2)
**Hypertension (%)**	(22.0)	(72.2)	(19.4)	(68.1)
SBP (mmHg)	122.4 ± 15.2	126.5 ± 16.5	121.4 ± 15.4	127.1 ± 16.3
DBP (mmHg)	76.2 ± 10.1	77.4 ± 10.9	75.6 ± 10.3	78.0 ± 10.4
**Diabetes (%)**	(6.1)	(19.1)	(6.2)	(17.8)
Fasting blood glucose (mg/dL)	97.6 ± 24.0	105.7 ± 32.8	97.8 ± 25.6	103.8 ± 29.6
**CKD (%)**	(6.3)	(18.8)	(4.0)	(20.6)
eGFR (mL/min)	88.0 ± 21.3	77.2 ± 21.7	93.4 ± 19.9	75.5 ± 21.2
**Dyslipidemia (%)**	(18.9)	(54.5)	(19.7)	(46.5)
Total cholesterol (mmol/L)	195.4 ± 37.0	185.6 ± 38.8	193.3 ± 37.2	190.3 ± 39.1
Triglyceride (mmol/L)	131.8 ± 90.2	131.4 ± 78.3	124.9 ± 84.3	138.4 ± 84.9
HDL cholesterol (mmol/L)	56.1 ± 25.7	53.1 ± 24.6	56.3 ± 17.9	55.2 ± 38.2
LDL cholesterol (mmol/L)	114.2 ± 38.0	107.3 ± 37.0	112.4 ± 34.3	109.8 ± 37.5
Previous MI (%)	(0.9)	(9.9)	(0.9)	(7.7)
Peripheral vascular disease (%)	(3.1)	(19.4)	(3.2)	(15.1)
Heart failure (%)	(2.1)	(27.5)	(2.1)	(23.0)
Previous stroke/TIA (%)	(3.6)	(20.6)	(3.6)	(17.3)
COPD	(2.2)	(10.7)	(2.3)	(9.8)
**Other blood test results**				
Hemoglobin (g/dL)	13.9 ± 1.6	13.7 ± 1.8	13.9 ± 1.7	13.8 ± 1.7
Aspartate transaminase (IU/L)	25.3 ± 16.3	27.1 ± 15.5	24.9 ± 17.1	27.8 ± 17.8
Alanine transaminase (IU/L)	25.0 ± 21.7	24.4 ± 20.2	24.1 ± 22.4	25.5 ± 18.9
Gamma-glutamyl transferase (U/L)	36.0 ± 49.3	45.7 ± 72.9	36.0 ± 53.9	46.4 ± 67.9
**Medications at enrollment**				
Antiplatelet agent (%)	(9.7)	(49.2)	(8.7)	(44.5)
Beta-blocker (%)	(7.5)	(40.0)	(6.7)	(34.2)
Statin (%)	(8.1)	(29.9)	(8.3)	(23.3)
Average PM_2.5_ concentration (μg/m^3^)	18.5	32.7	18.5	35.7
Total follow-up year	3.8	3.9	3.8	3.9

*AF* atrial fibrillation, *BMI* body mass index (kg/m^2^), *CKD* chronic kidney disease (eGFR lower than 60 mL/min estimated by serum creatinine using CKD-EPI formula)^37^, *COPD* chronic obstructive pulmonary disease, *DBP* diastolic blood pressure, *eGFR* estimated glomerular filtration rate (mL/min), *HDL* high density lipoprotein, *LDL* low density lipoprotein, *MI* myocardial infarction, *PM*_*2.5*_ particulate matter < 2.5 μm in diameter, *SBP* systolic blood pressure, *TIA* transient ischemic attack.

*Socioeconomic status was divided into two groups: higher (≥ 51% of income level) and lower (< 51% of income level).

### Application of PM_2.5_ to traditional regression analysis improves the prediction of incident AF

The air pollution and meteorological measurements are described in detail in Supplementary Results (Supplementary Table 3). We have previously reported on the association between increased exposure to long-term average PM_2.5_ and increased incidence of AF^8^. The traditional regression analysis with a forward selection method revealed PM_2.5_ and the following seven clinical risk factors to be associated with incident AF: older age (odds ratio [OR] 1.06 [1.05–1.07] per year increase, p < 0.001), male sex (OR 1.57 [1.42–1.73], p < 0.001), higher BMI (OR 1.04 [1.02–1.05] per 1 kg/m^2^ increase, p < 0.001), higher diastolic blood pressure (OR 1.05 [1.03–1.8] per 5 mmHg increase, p = 0.013) and systolic blood pressure (OR 1.02 [1.01–1.04] per 5 mmHg increase, p = 0.034), history of previous heart failure (OR 2.20 [1.90–2.54], p < 0.001), lower serum estimated glomerular filtration rate (OR 1.04 [1.02–1.07] per 10 mL/min decrease, p < 0.001), and higher level of PM_2.5_ exposure (OR 1.27 [1.24–1.31] per 10 μg/m^3^ increase, p < 0.001). After the application of PM_2.5_ to the readily available clinical risk factors, the c-index of the model for predicting incident AF was improved from 0.643 (0.636–0.649) to 0.819 (0.813–0.825) (p < 0.001) (Table 2). The NRI and IDI between these models were also positive (1.069 [1.038–1.103] and 0.302 [0.294–0.322], respectively) (Table 2).

**Table 2:** Performance of predictive models for incident AF risk during follow-up period in overall general population.

Models	c-index (95% CI)	NRI	IDI
**Traditional regression analysis (TR)**			
Clinical variables-adjusted (TR1, model 1)*	0.643 (0.636–0.649)	Ref	Ref
TR1 plus PM_2.5_-adjusted (model 1)^†^	0.819 (0.813–0.825)	1.069 (1.038–1.103)	0.302 (0.294–0.322)
Clinical variables-adjusted (TR2, model 2)*	0.684 (0.675–0.693)	Ref	Ref
TR2 plus PM_2.5_-adjusted (model 2)^†^	0.869 (0.862–0.876)	1.087 (1.060–1.113)	0.219 (0.209–0.228)
CHA_2_DS_2_-VASc score	0.654 (0.646–0.661)	Ref	Ref
PM-CHA_2_DS_2_-VASc score^‡^	0.859 (0.848–0.870)	1.078 (1.059–1.102)	0.220 (0.208–0.233)
CHADS_2_ score	0.652 (0.646–0.657)	Ref	Ref
PM-CHADS_2_ score^‡^	0.823 (0.810–0.836)	0.981 (0.962–1.001)	0.042 (0.029–0.054)
HATCH score	0.669 (0.661–0.676)	Ref	Ref
PM-HATCH score^‡^	0.849 (0.837–0.860)	1.004 (0.983–1.024)	0.053 (0.042–0.064)
**Machine learning models**			
Support vector machine			
Clinical variables-adjusted*	0.766 (0.757–0.775)	Ref	Ref
Clinical variables plus PM_2.5_-adjusted^†^	0.903 (0.895–0.910)	1.061 (1.038–1.083)	0.270 (0.260–0.281)
Decision tree			
Clinical variables-adjusted*	0.801 (0.787–0.815)	Ref	Ref
Clinical variables plus PM_2.5_-adjusted^†^	0.931 (0.925–0.937)	1.054 (1.027–1.081)	0.265 (0.256–0.275)
Random forest			
Clinical variables-adjusted*	0.838 (0.830–0.846)	Ref	Ref
Clinical variables plus PM_2.5_-adjusted^†^	0.939 (0.933–0.945)	1.027 (1.006–1.050)	0.242 (0.232–0.253)
Naïve Bayes			
Clinical variables-adjusted*	0.833 (0.825–0.841)	Ref	Ref
Clinical variables plus PM_2.5_-adjusted^†^	0.894 (0.888–0.900)	0.987 (0.959–1.014)	0.152 (0.142–0.162)
Deep neural network			
Clinical variables-adjusted*	0.813 (0.800–0.826)	Ref	Ref
Clinical variables plus PM_2.5_-adjusted^†^	0.849 (0.834–0.865)	0.792 (0.745–0.837)	0.088 (0.074–0.101)
Extreme gradient boosting			
Clinical variables-adjusted*	0.845 (0.837–0.853)	Ref	Ref
Clinical variables plus PM_2.5_-adjusted^†^	0.954 (0.949–0.959)	1.277 (1.218–1.334)	0.461 (0.438–0.485)

*AF* atrial fibrillation, *BMI* body mass index, *CI* confidence interval, *DBP* diastolic blood pressure, *eGFR* estimated glomerular filtration rate, *HF* heart failure, *IDI* integrated discrimination improvement index, *NRI* category-free net reclassification improvement index, *PM*_*2.5*_ particulate matter < 2.5 μm in diameter, *SBP* systolic blood pressure, *TR* traditional regression analysis.

*TR1 (model 1), clinical 6 variables (age, sex, BMI, SBP, previous HF history, and serum eGFR) -adjusted c-index; TR2 (model 2), clinical 12 variables (age, sex, BMI, SBP, diabetes, previous HF history, previous stroke/TIA history, previous myocardial infarction history, serum eGFR, serum total cholesterol, smoking history, and alcohol intake habit) -adjusted c-index. DBP was not included in these models because of multicollinearity with SBP.

^†^Clinical 6 variables (*) plus PM_2.5_-adjusted c-index.

^‡^We pointed as 1 if PM_2.5_ ≥ 15 μg/m^3^ based on Korean National Ambient Air Quality Standards and added this PM score to established CHADS_2_, CHA_2_DS_2_-VASc, and HATCH scores as PM-CHADS_2_, PM-CHA_2_DS_2_-VASc, PM-HATCH scores^25^.

### Risk factors including PM_2.5_ for incident AF and score development

Crude and multivariable-adjusted Cox regression analyses showed that heart failure, hypertension, age (≥ 75 years), diabetes, previous stroke/transient ischemic attack, previous myocardial infarction or peripheral vascular disease, male sex, chronic obstructive pulmonary disease (these are components of the CHA_2_DS_2_-VASc, CHADS_2_, and HATCH scores), and PM_2.5_ (≥ 15 μg/m^3^ based on the Korean annual NAAQS)^25^ were independent risk factors for incident AF (Table 3). According to these multivariable Cox regression analyses, PM_2.5_ ≥ 15 μg/m^3^ (HRs 1.1–1.4) was pointed as 1 compared with HRs of other clinical variables and added this to existing CHA_2_DS_2_-VASc, CHADS_2_, and HATCH systems, then we developed new simple PM-CHA_2_DS_2_-VASc, PM-CHADS_2_, and PM-HATCH scores (Table 3). The total scores ranged from 0 to 10, 0 to 7, and 0 to 8 points, respectively.

**Table 3:** Association of PM_2.5_ with the incidence of AF in overall general population.

Variables	Crude*	Adjusted for clinical variables^†^ including CHA_2_DS_2_-VASc score components plus PM_2.5_	Adjusted for clinical variables^†^ including CHADS_2_ score components plus PM_2.5_	Adjusted for clinical variables^†^ including HATCH score components plus PM_2.5_
	HR (95% CI)	HR (95% CI)	HR (95% CI)	HR (95% CI)
PM_2.5_ (≥ 15 μg/m^3^)^‡^	1.439 (1.231–1.623) ||	1.248 (1.103–1.384) ||	1.186 (1.122–1.251) ||	1.329 (1.189–1.466) ||
**Age** §				
≥ 75 years	6.548 (5.995–7.152) ||		2.167 (1.915–2.444) ||	2.115 (1.873–2.381) ||
65–74; ≥ 75 years	5.670 (5.262–6.109) ||	3.402 (3.127–3.701) ||		
Male sex	1.352 (1.263–1.447) ||	1.634 (1.526–1.750) ||		
Heart failure	6.775 (6.124–7.494) ||	2.013 (1.806–2.243) ||	2.102 (1.885–2.343) ||	2.612 (2.403–2.846) ||
Hypertension	5.196 (4.855–5.560) ||	1.743 (1.524–1.980) ||	1.931 (1.638–2.247) ||	2.074 (1.778–2.392) ||
Diabetes	3.178 (2.908–3.473) ||	1.310 (1.202–1.430) ||	1.364 (1.241–1.498) ||	
Stroke/TIA	4.368 (3.965–4.812) ||	2.282 (2.156–2.422) ||	2.495 (2.348–2.659) ||	2.503 (2.355–2.667) ||
Vascular disease	3.491 (3.081–3.955) ||	1.285 (1.142–1.449) ||		
COPD	4.445 (3.954–4.997) ||			1.780 (1.574–2.014) ||

*AF* atrial fibrillation, *CI* confidence interval, *HR* hazard ratio, *PM*_*2.5*_ particulate matter < 2.5 μm in diameter, *TIA* transient ischemic attack.

*Unadjusted Cox proportional hazards model.

^†^Cox proportional hazards model adjusted for clinical variables. Clinical variables were remaining CHA_2_DS_2_-VASc, CHADS_2_, and HATCH components of age, male sex, heart failure, hypertension, diabetes, stroke/transient ischemic attack, vascular disease including previous history of myocardial infarction or peripheral vascular disease, and chronic obstructive pulmonary disease.

^‡^In these Cox proportional hazards models, PM_2.5_ variable was analyzed as categorical variable with dividing subjects into subgroups as PM_2.5_ ≥ 15 μg/m^3^ or < 15 μg/m^3^ based on the Korean National Ambient Air Quality Standards^25^.

^§^Age variable was analyzed as binary or three categorical variables with dividing subjects into subgroups as: (for binary categorical variables) age ≥ 75 or < 75 years; or (for three categorical variables) age ≥ 75, 65–74, or < 65 years.

|| p-value < 0.001.

The scores showed good discrimination with c-indices of 0.859 (0.848–0.870), 0.823 (0.810–0.836), and 0.849 (0.837–0.860), respectively (Table 2). These scoring systems showed significantly better performances for predicting incident AF than each existing score (CHA_2_DS_2_-VASc, CHADS_2_, and HATCH), and their NRI and IDI were also positive (Table 2).

### Incidence rates of AF and the PM-CHA_2_DS_2_-VASc, PM-CHADS_2_, and PM-HATCH scores

**Figure 2:**
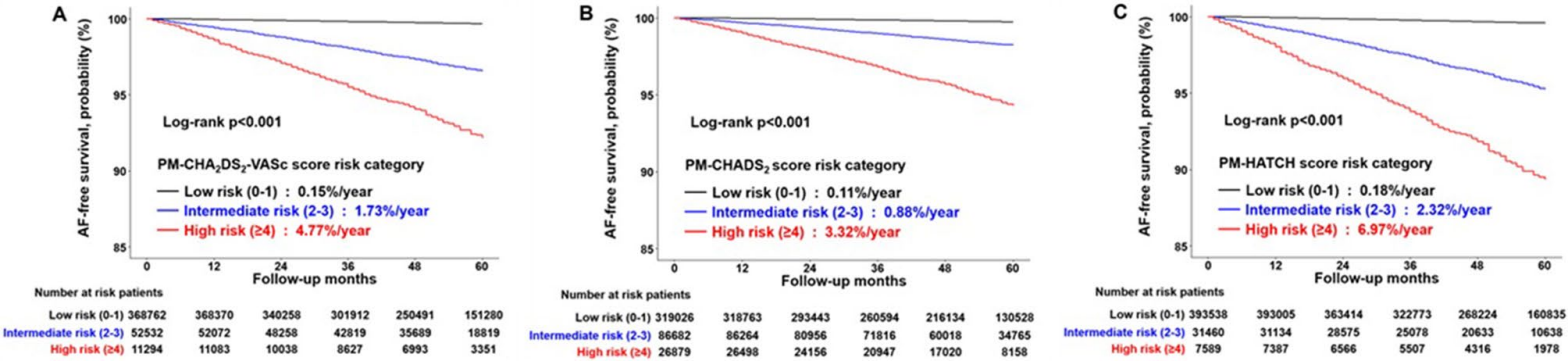
Kaplan–Meier curves for risk categories according to PM-CHA_2_DS_2_-VASc, PM-CHADS_2_, and PM-HATCH scores. Patients were divided into three groups as low (0–1 points), intermediate (2–3 points), and high risk (≥ 4 points) groups. We pointed as 1 if PM_2.5_ ≥ 15 μg/m^3^ based on Korean National Ambient Air Quality Standards.

Supplementary Table 4 shows the annual incidence rates (%/year) and hazard ratios at each point of these scoring systems. We divided the overall population into three groups according to each score: low risk (0–1 point; 0.13%/year by PM-CHA_2_DS_2_-VASc score, 0.11%/year by PM-CHADS_2_ score, and 0.17%/year by PM-HATCH score), intermediate risk (2–3 points; 1.30%/year by PM-CHA_2_DS_2_-VASc score, 0.70%/year by PM-CHADS_2_ score, and 1.75%/year by PM-HATCH score), and high risk (≥ 4 points; 2.74%/year by PM-CHA_2_DS_2_-VASc score, 2.05%/year by PM-CHADS_2_ score, and 3.51%/year by PM-HATCH score). Kaplan–Meier curves showed consistent findings according to risk categories (log-rank p < 0.001, Fig. 2).

### Comparing ML models with the traditional regression model

To estimate the crude accuracies of the ML models for predicting incident AF, we compared the six ML models and the traditional regression analysis model with age, sex, and BMI as input variables (Table 4). The c-indices of the six ML models (SVM, decision tree, random forest, naïve Bayes, deep neural network, and extreme gradient boosting models) were 0.699 (0.688–0.710), 0.786 (0.771–0.800), 0.787 (0.772–0.801), 0.794 (0.780–0.807), 0.779 (0.768–0.790), and 0.790 (0.776–0.805), respectively, and they were all significantly better than the c-index of the traditional regression analysis model (c-index 0.604 [0.598–0.611], all p < 0.001). All NRI and IDI were also positive (Table 4). The extreme gradient boosting model showed the highest c-index for predicting incident AF among these models (Table 4).

**Table 4:** Performance of predictive models for incident AF risk during follow-up period in overall general population (age, sex, and BMI-adjusted models).

Predictive models*	c-index (95% CI)	NRI (95% CI)	IDI (95% CI)
Traditional regression analysis	0.604 (0.598–0.611)	Ref	Ref
**Machine learning models**			
Support vector machine	0.699 (0.688–0.710)	0.280 (0.220–0.340)	0.002 (0.001–0.003)
Decision tree	0.786 (0.771–0.800)	0.806 (0.747–0.866)	0.010 (0.009–0.011)
Random forest	0.787 (0.772–0.801)	0.764 (0.701–0.827)	0.006 (0.005–0.007)
Naïve Bayes	0.790 (0.776–0.805)	0.792 (0.732–0.853)	0.009 (0.008–0.010)
Deep neural network	0.779 (0.768–0.790)	0.218 (0.182–0.253)	0.002 (0.001–0.003)
Extreme gradient boosting	0.794 (0.780–0.807)	0.536 (0.484–0.589)	0.005 (0.004–0.006)

*AF* atrial fibrillation, *BMI* body mass index, *CI* confidence interval, *IDI* integrated discrimination improvement index, *NRI* category-free net reclassification improvement index.

*Age, sex, and BMI were used for constructing these predictive models (age, sex, and BMI were adjusted for traditional regression analysis, and these variables were used as input variables for training the listed machine learning models).

### Validation by ML models and the application of PM_2.5_ for predicting incident AF

We used several ML models and performed analyses for predicting incident AF in our cohort. We used the 27 variables listed in Table 1 as input variables for the ML models and performed training using a discovery cohort of 302,811 subjects including 2444 with incident AF (0.8%) that developed over the 5-year follow-up period (Table 1). The feature selection process revealed variables that were most relevant for building the ML models (Supplementary Figure or Variable importance 10 ranking—Table 5). For random forest and extreme gradient boosting ML models, based on decision trees, the rank variable importance is determined by the selection frequency of the variables as a decision node, whereas SVM uses the sensitivity of generalization error bounds with respect to a variable and neural networks use overall weighting of the variable within the model^26^. PM_2.5_ was also highly ranked and other variables were also selected, as described in Supplementary Figure and Table 5.

**Table 5:** Ranking of the 10 most important variables for algorithms run for predicting incident AF (among 27 clinical variables).

Ranking of variables	Traditional regression analysis	Support vector machines with linear Kernel	Decision tree	Random forest	Extreme gradient boosting
1	Heart failure	Heart failure	Age	Serum eGFR	Heart failure
2	Systolic blood pressure	Systolic blood pressure	Serum eGFR	Systolic blood pressure	Systolic blood pressure
3	Age	Age	Heart failure	Age	Age
4	Previous ischemic stroke/TIA	Previous ischemic stroke/TIA	Systolic blood pressure	Heart failure	PM_2.5_
5	PM_2.5_	PM_2.5_	Previous ischemic stroke/TIA	PM_2.5_	Serum triglyceride
6	Serum eGFR	Serum eGFR	PM_2.5_	Sex	Serum total cholesterol
7	Serum triglyceride	Previous MI	Serum triglyceride	BMI	Serum HDL cholesterol
8	Sex	Sex	Sex	Smoking history	BMI
9	Smoking history	Smoking history	Smoking history	Fasting blood glucose	Serum eGFR
10	Serum total cholesterol	BMI	BMI	Previous ischemic stroke/TIA	Serum LDL cholesterol

*AF* atrial fibrillation, *BMI* body mass index, *CI* confidence interval, *eGFR* estimated glomerular filtration rate (mL/min), *HDL* high density lipoprotein, *HR* hazard ratio, *LDL* low density lipoprotein, *MI* myocardial infarction, *PM*_*2.5*_ particulate matter < 2.5 μm in diameter, *TIA* transient ischemic attack.

**Figure 3:**
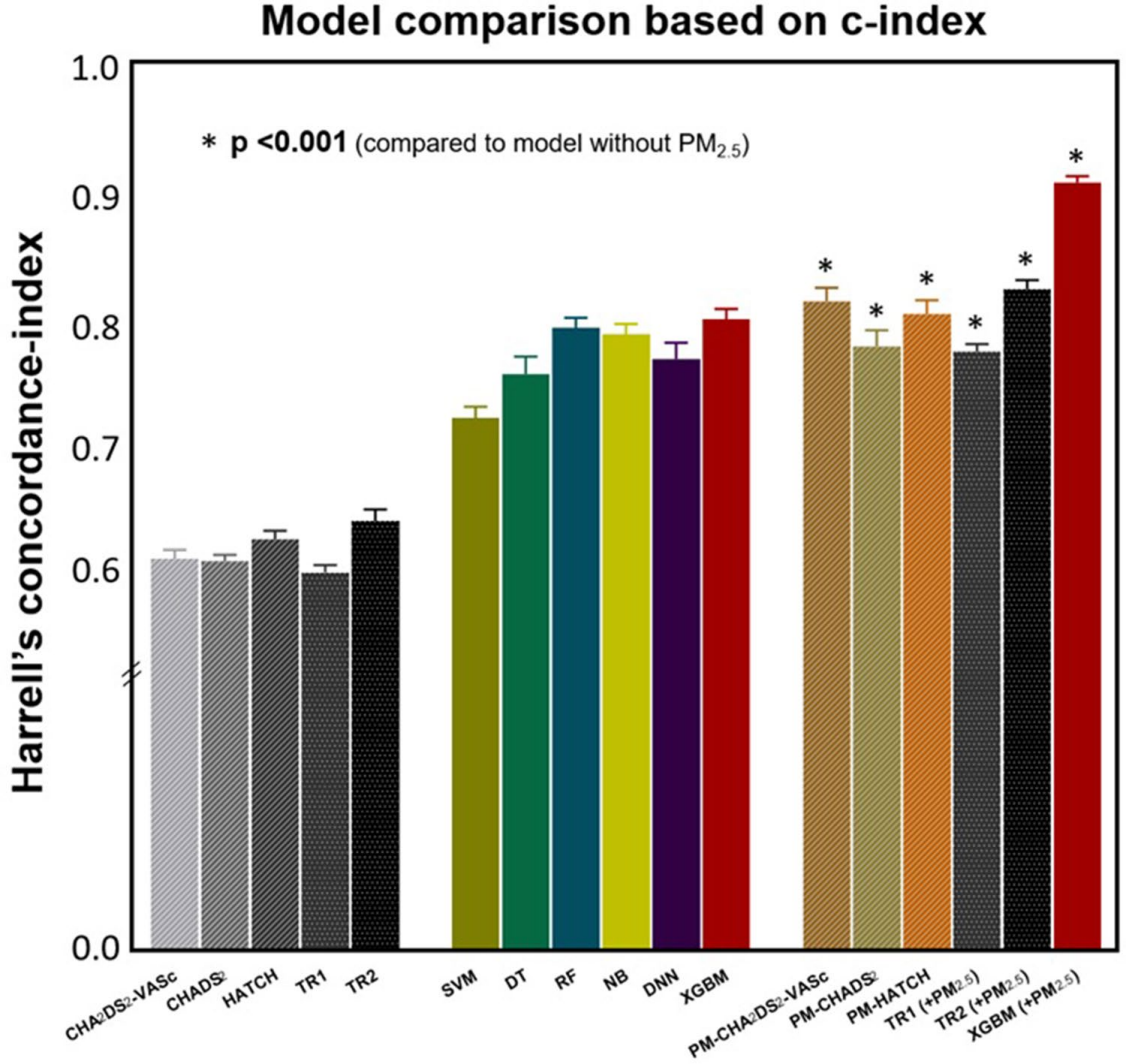
Comparison of models for predicting incident atrial fibrillation based on the c-index. *DNN* deep neural network model, *DT* decision tree model, *NB* naïve Bayes model, *PM*_*2.5*_ particulate matter < 2.5 μm in diameter, *RF* random forest model, *SVM* support vector machine, *TR1* traditional regression analysis model using clinical six variables (adjusted variables were same with that of TR1 (model 1) in Table 2) as input variables, *TR2* traditional regression analysis model using clinical 12 variables (adjusted variables were same with that of TR2 (model 2) in Table 2), *XGBM* extreme gradient boosting model.

After applying the tenfold cross-validation algorithm, the best ML model was the extreme gradient boosting model of the boosted ensemble algorithm with a c-index of 0.845 (0.837–0.853) (Table 2). After adding PM_2.5_ as an input variable, the performance of predicting incident AF was improved, with a c-index of 0.954 (0.949–0.959) with NRI of 1.277 (1.218–1.334) and IDI of 0.461 (0.438–0.485) (Table 2). For models using SVM, decision tree, random forest, naïve Bayes, and deep neural network algorithms, the performances for predicting incident AF were significantly improved after applying PM_2.5_, with c-indices of 0.903 (0.895–0.910), 0.931 (0.925–0.937), 0.939 (0.933–0.945), 0.894 (0.888–0.900), and 0.849 (0.834–0.865), respectively (Fig. 3 and Table 2). All NRI and IDI were also positive (Table 2).

## Discussion

There have been few studies about the correlations between PM_2.5_ and incident AF, and the clinical significance of PM_2.5_ for predicting AF incidence has not been investigated. In this study, we investigated the clinical impact of PM_2.5_ on predicting AF incidence by using ML methods in the general population of an Asian country affected by high air pollution levels. ML methods identified the clinically important variables for predicting incident AF, and PM_2.5_ was also identified as a highly ranked important variable. With the addition of the PM_2.5_ variable, the prediction performance significantly improved with both traditional regression analysis and ML methods. Additionally, based on traditional regression analysis, we constructed scoring systems for predicting incident AF by adding the PM_2.5_ variable to existing risk prediction systems: PM-CHA_2_DS_2_-VASc, PM-CHADS_2_, and PM-HATCH, which showed better predicting performances than the established scoring systems.

Although some studies, including our previous study^8^, have shown the relationships between air pollution exposure and AF development in patients with known cardiac diseases^27^, some studies from Western countries did not show a relationship between PM_2.5_ exposure and incident AF^11,12^. However, these studies were performed in European countries and the United States, where the air pollution levels were much lower than the levels in Asian countries. Therefore, the effect sizes could be low in those studies. In our nationwide dataset, to facilitate the data-driven analysis for revealing AF risk factors, we used 27 readily available parameters among the general population, and PM_2.5_ was identified as a highly ranked variable. Adding the information of PM_2.5_ exposure to known clinical risk factors can enable a better prediction of incident AF in the general population. Additionally, we attempted to apply this information about PM_2.5_ exposure for predicting incident AF in clinical practice by constructing relevant risk scores based on the Korean NAAQS, which might add some information when managing patients with AF risk factors. Further prospective studies using these new risk scoring systems will be needed whether upstream medical therapy is beneficial to prevent incident AF in the general population.

The adverse health effects related to air pollution have been studied since 1993^28^, even for arrhythmias^29^. One suggested mechanism is the occurrence of myocardial repolarization abnormalities contributing to arrhythmias^30^ caused by systemic inflammatory cytokines produced by pulmonary inflammatory responses after inhaling particles^6^. Another suggested mechanism includes alteration of the cardiac autonomic nervous system that occurs with the inhalation of particles mediated by reactive oxygen species^31,32^, these adverse inhalation effects can be diminished in patients with chronic lung parenchymal diseases^33,34^.

As an advanced computing technology for artificial intelligence, ML is increasingly used in cardiology to meaningfully process data that exceed the capacity of the human brain^35^. Unlike traditional statistical analyses, ML models can accept enormous data as input variables and can improve the prediction performances through a repetitive training process, thus offering more applicable prediction models to external datasets^20,36^. Everyone can access this advanced computing technology and it can rapidly construct models by automatically training process, consequently it can offer better prediction model than traditional model manually made by human^35^. However, ML is highly data-dependent (“garbage-in, garbage-out”) and hard to interpret, and it often develops overfitting problems^35^.

To the best of our knowledge, this is the first cohort study with 1,666,528 person-years of follow-up to assess the prediction performance of long-term PM_2.5_ exposure for incident AF. Additionally, long-term PM_2.5_ exposure was identified as a highly important variable for predicting incident AF by using ML methods. After adding the PM_2.5_ variable to established AF prediction scoring systems, the prediction performances for incident AF significantly improved.

Our study suggests that applying long-term average PM_2.5_ measurements in clinical practice could better predict the development of AF in patients. Additionally, ML using boosted ensemble methods can predict incident AF better, with readily available subject characteristics, than traditional regression analysis. The detailed characteristics of the subjects in this study allowed blood pressure measurements, blood test results including fasting glucose and cholesterol profiles, and smoking and alcohol intake habits to be integrated in these analyses.

On the basis of these findings, we constructed scoring systems for predicting incident AF by adding the PM_2.5_ variable to existing risk prediction approaches: PM-CHA_2_DS_2_-VASc, PM-CHADS_2_, and PM-HATCH, which showed better predicting performances than established scoring systems.

Although our findings from traditional regression analysis and novel ML methods drew similar results, there are some limitations. Although we set disease-free baseline period (7 years: 2002–2008) with excluding subjects with a previous AF history, the existence of selection bias cannot be ruled out. However, the diagnostic accuracy of AF with this manner was previously validated in our NHIS database^17^. Although we excluded subjects who changed residence within the study period, subjects’ air pollutant exposure or specific locations could not be fully reflected during the period. In addition, as our data were from the National Health Insurance administrative claims database, the exact hour of AF development could not be identified. Therefore, we thought that the analysis for the effects of acute exposure might draw somewhat biased results, and further investigations are needed^8^. Although we used previously established risk scoring systems such as CHA_2_DS_2_-VASc^4^, CHADS_2_^3^, and HATCH^5^ scores, they were not originally designed for predicting incident AF. However, these scores included important clinical comorbidities affect AF development, and we also assessed the prediction performances of traditional regression analyses and ML methods using readily available clinical risk factors and it showed better prediction performances with PM_2.5_ than those without PM_2.5_ (Table 2 and Fig. 3). Our prediction models did not adjust some confounders such as echocardiographic parameters (left atrial size, left ventricular ejection fraction, and ventricular chamber size), other chronic diseases (liver diseases, dementia, and chronic systemic inflammatory diseases), and some exposure confounders (occupational aspects who usually work inside or outside) that affect AF development or air pollution exposures. As we did not investigate the associations between air pollution and myocardial repolarization or inflammatory markers, the mechanism behind the relationship of exposure to air pollution and AF remains unclear. Although statistical approaches including cross-validation to minimize overfitting were applied when constructing ML models, and they supported our main results, external validation is needed especially in Western countries where the air pollution level is low.

## Conclusions

Data-driven approaches suggested long-term exposure to PM_2.5_ air pollution as a risk factor robustly associated with incident AF. Such ML models combining readily available clinical characteristics and PM_2.5_ measurements were found to predict incident AF better than traditional statistical models or even established risk prediction approaches in the Korean general population exposed to high levels of air pollution. Further external validation is warranted especially in Western countries affected by low levels of air pollution.

## Supplementary information

Supplementary Information.
