# Supplementary material for: Long-term PM2.5 exposure and the clinical application of machine learning for predicting incident atrial fibrillation
Source: Sci Rep. 2020 Oct 1;10:16324. doi: 10.1038/s41598-020-73537-8 (PMC7530980; doi:10.1038/s41598-020-73537-8)
Supplement: Supplementary file 1 — Supplementary Information. [file 41598_2020_73537_MOESM1_ESM.docx]

**Long-term PM_2.5_ exposure and the clinical application of machine learning for predicting incident atrial fibrillation**

In-Soo Kim, MD^1,2^; Pil-Sung Yang, MD^3^; Eunsun Jang, MS^1^; Hyunjean Jung, MD^1^; Seng Chan You, MD^4^; Hee Tae Yu, MD, PhD^1^; Tae-Hoon Kim, MD^1^; Jae-Sun Uhm, MD, PhD^1^; Hui-Nam Pak, MD, PhD^1^; Moon-Hyoung Lee, MD, PhD^1^; Jong-Youn Kim, MD, PhD^2^*; Boyoung Joung, MD, PhD^1^*

*^1^Division of Cardiology, Department of Internal Medicine, Severance Cardiovascular Hospital, Yonsei University College of Medicine, Seoul, Republic of Korea.*

*^2^Division of Cardiology, Department of Internal Medicine, Gangnam Severance Hospital, Yonsei University College of Medicine, Seoul, Republic of Korea.*

*^3^Department of Cardiology, CHA Bundang Medical Center, CHA University, Seongnam, Republic of Korea.*

*^4^Department of Biomedical Informatics, Ajou University School of Medicine, Suwon-si, Gyeonggi-do, Republic of Korea.*

[*Joint senior authors]

**Short title**: PM_2.5_ and incident AF predict by machine learning

Total word count: 2,614

Total number of Tables: 5

Total number of Figures: 3

Total number of Supplementary Tables: 4

Total number of Supplementary Figure: 1

**Address for correspondence:**

Jong-Youn Kim, MD, PhD.

Associate Professor, Division of Cardiology, Department of Internal Medicine

Gangnam Severance Hospital, Yonsei University College of Medicine

211 Eonju-ro Gangnam-gu, Seoul, Korea, 06273

Telephone: 82-2-2019-3307, Fax: 82-2-3463-3882

Email: [jykim0706@yuhs.ac](mailto:jykim0706@yuhs.ac)

Boyoung Joung, MD, PhD.

Professor, Division of Cardiology, Department of Internal Medicine

Yonsei University College of Medicine

50-1 Yonsei-ro, Seodaemun-gu, Seoul, Republic of Korea 03722

Phone: +82-2-2228-8460, FAX: +82-2-393-2041

Email: [cby6908@yuhs.ac](mailto:cby6908@yuhs.ac)

**Supplementary Materials**

**Supplementary Results**

**Supplementary References**

**Supplementary Figure**

**Supplementary Tables 1-4**

**Supplementary Results**

***Air pollution and meteorological measurements***

PM_2.5_ and meteorological variables were measured on more than 95% of the follow-up days as described in Supplementary Table 3. Temperature and humidity were not correlated with PM_2.5_.

**Supplementary References**

1. Levey, A. S. *et al.* A new equation to estimate glomerular filtration rate. *Ann Intern Med* **150**, 604-612 (2009).

2. Annual Report of Air Quality in Korea, National Institute of Environmental Research, Republic of Korea <http://library.me.go.kr/search/DetailView.Popup.ax?sid=4&cid=5618423> (2018).

**Supplementary Figure.** Feature selection using extreme gradient boosting model. Readily available 27 clinical risk factors including PM_2.5_ were considered as input variables. Backward selection with this boosted ensemble model was used to identify the model with the highest c-index. Rank variable importance was determined by the selection frequency of the variables as a decision node. AST, aspartate transaminase; BMI, body mass index; COPD, chronic obstructive pulmonary disease; eGFR, estimated serum glomerular filtration rate (mL/min); GGT, gamma-glutamyl transferase; HDL, high-density lipoprotein; HF, heart failure; MI, myocardial infarction; PM_2.5_, particulate matter <2.5μm in diameter; SBP, systolic blood pressure; TIA, transient ischemic attack.

**
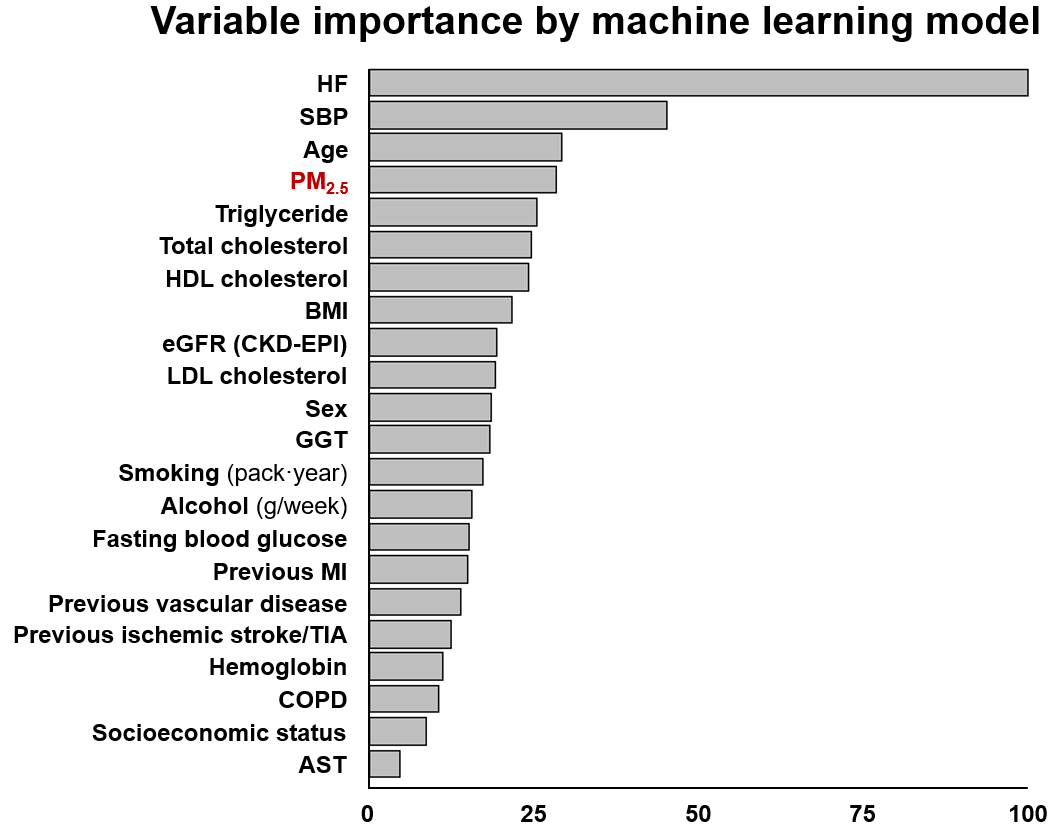
**

**Supplementary Table 1**. Definitions of comorbidities and its ICD-10 codes or conditions.

| **Comorbidities** | **Definitions** | **ICD-10 codes or conditions** |
| --- | --- | --- |
| AF | Defined from the diagnosis ^a^ | ICD-10: I48 |
| Non-valvular AF | Defined from AF patients with absence of diagnoses such as mitral valve stenosis or prosthetic valve disease | AF patients (I48) without ICD-10 codes of such diseases (I050, I052, I342) |
| Heart failure | Defined from the diagnosis ^a^ | ICD-10: I110, I50 |
| Hypertension | Defined from the diagnosis ^a^ | ICD-10: I10 |
| Diabetes mellitus | Defined from the diagnosis ^a^ plus treatment | ICD-10: E10, E11, E12, E13, E14  Treatment: all kinds of oral antidiabetics or insulin. |
| Stroke | Defined from admission diagnosis plus imaging studies | Any admission diagnosis with ICD-10 code of I60, I61, I62, I63, or I64 with concomitant brain imaging studies (including computed tomography or magnetic resonance imaging). |
| Myocardial infarction | Defined from the diagnosis ^a^ | ICD-10: I21, I22, I23 |
| Peripheral vascular disease | Defined from the diagnosis ^a^ | ICD-10: I71, I72, I73, I74 |
| Malignant disease | Defined from the diagnoses of any malignant disease | ICD-10: C00-C97 |
| Cirrhotic liver disease | Defined from the diagnosis ^a^ | ICD-10: K74 |
| Chronic kidney disease (eGFR <60 mL/min) | Defined from the diagnosis or laboratory results of health examination: eGFR lower than 60 mL/min estimated by serum creatinine using CKD-EPI formula[^1^](#_ENREF_1) | ICD-10: N18, N19; or laboratory results of health examination (eGFR <60 mL/min). |
| Chronic obstructive pulmonary disease | Defined from the diagnosis ^a^ plus treatment | J41, J42, J43 (except J430), J44  Treatment: SABA, SAMA, LABA, LAMA, ICS, ICS+LABA, or methylxanthine (>1 months). |

AF, atrial fibrillation; CKD-EPI, chronic kidney disease-epidemiology collaboration; eGFR, estimated glomerular filtration rate; ICD-10 code, the 10^th^ revision of the International Classification of Disease; ICS, inhaled corticosteroid; LABA, long acting beta-agonist; LAMA, long acting muscarinic antagonist; SABA, short acting beta-agonist; SAMA, short acting muscarinic antagonist.

^a^, To ensure accuracy, comorbidities were established based on the diagnosis of ICD-10 codes by the first inpatient diagnosis or outpatient diagnosis of two different days.

**Supplementary Table 2.** Korean National Air Quality Standards for PM_2.5_.[^2^](#_ENREF_2)

| **Air pollutant** | **Republic of Korea** | **United States** | **European Union** | **Measurement method** |
| --- | --- | --- | --- | --- |
| PM_2.5_ | 35 ㎍/m^3^ (24 hour)  15 ㎍/m^3^ (annual) | 35 ㎍/m^3^ (24 hour)  12 ㎍/m^3^ (annual) | 25 ㎍/m^3^ (annual) | Laser photometry |

CO, carbon monoxide; NO_2_, nitrogen dioxide; O_3_, ozone; PM_2.5_, particulate matter <2.5㎛ in diameter; PM_10_, particulate matter <10㎛ in diameter; ppm, parts per million; SO_2_, sulfur dioxide; UV, ultraviolet.

**Supplementary Table 3**. Distribution summary and Pearson’s correlation coefficients of daily average PM_2.5_ concentration and meteorological variables during the study period from January 1, 2009, to 31 December 31, 2013.

| **Variables** | **Distribution summary** | | | | | |  | **Pearson’s correlation coefficients, r** |
| --- | --- | --- | --- | --- | --- | --- | --- | --- |
|  | **Measured days (%)** | **Min** | **25^th^** | **50^th^** | **75^th^** | **Max** |  | **PM_2.5_** |
| **Air pollutant** |  |  |  |  |  |  |  |  |
| PM_2.5_, μg/m^3^ | 96.7 | 1.7 | 19.9 | 32.2 | 44.4 | 336.0 |  | 1.00 |
| **Meteorological variables** |  |  |  |  |  |  |  |  |
| Temperature, ˚C | 99.7 | -15.7 | 7.3 | 13.8 | 20.2 | 33.0 |  | -0.11 |
| Humidity, % | 99.4 | 11.6 | 52.3 | 63.2 | 74.1 | 100.0 |  | 0.03 |

PM_2.5_, particulate matter <2.5㎛ in diameter.

**Supplementary Table 4.** Annual incidence of AF according to PM-CHA_2_DS_2_-VASc, PM-CHADS_2_, and PM-HATCH scores.

| **Scores** * | **No. of subjects** | **No. of incidents of AF** | **Incidence of AF**  **(%/year)** | **Adjusted HR** † | **95% CI** |
| --- | --- | --- | --- | --- | --- |
| **PM-CHA_2_DS_2_-VASc** |  |  |  |  |  |
| **0** | 273,332 | 367 | 0.04 | 1.000 (Ref) | **-** |
| **1** | 92,867 | 1,509 | 0.41 | 1.030 | 1.006-1.052 |
| **2** | 37,691 | 1,475 | 1.01 | 1.122 | 1.057-1.191 |
| **3** | 16,180 | 1,208 | 1.97 | 1.197 | 1.027-1.395 |
| **4** | 7,948 | 656 | 2.21 | 1.210 | 1.034-1.415 |
| **5** | 3,580 | 392 | 3.01 | 1.218 | 1.025-1.447 |
| **≥6** | 989 | 217 | 6.28 | 1.264 | 1.084-1.473 |
| **PM-CHADS_2_** |  |  |  |  |  |
| **0** | 135,469 | 233 | 0.04 | 1.000 (Ref) | **-** |
| **1** | 181,090 | 1,044 | 0.15 | 1.040 | 1.013-1.068 |
| **2** | 60,231 | 1,254 | 0.53 | 1.077 | 1.057-1.095 |
| **3** | 27,119 | 1,112 | 1.06 | 1.125 | 1.099-1.153 |
| **4** | 14,884 | 884 | 1.57 | 1.160 | 1.062-1.269 |
| **5** | 7,680 | 579 | 2.03 | 1.214 | 1.090-1.340 |
| **≥6** | 6,114 | 718 | 3.31 | 1.282 | 1.060-1.551 |
| **PM-HATCH** |  |  |  |  |  |
| **0** | 291,209 | 519 | 0.05 | 1.000 (Ref) | **-** |
| **1** | 99,791 | 2,015 | 0.51 | 1.012 | 0.966-1.065 |
| **2** | 14,287 | 602 | 1.12 | 1.050 | 1.029-1.074 |
| **3** | 18,348 | 1,559 | 2.24 | 1.052 | 1.034-1.071 |
| **4** | 5,335 | 624 | 3.22 | 1.098 | 1.044-1.155 |
| **5** | 2,320 | 303 | 3.61 | 1.122 | 1.047-1.201 |
| **≥6** | 1,297 | 203 | 4.59 | 1.252 | 1.007-1.556 |

AF, atrial fibrillation; BMI, body mass index; CI, confidence interval; eGFR, estimated glomerular filtration rate; HDL, high density lipoprotein; HR, hazard ratio; LDL, low density lipoprotein; MI, myocardial infarction; PM_2.5_, particulate matter <2.5 μm in diameter; TIA, transient ischemic attack.

* In these scoring systems, we pointed as 1 if PM_2.5_ ≥15 μg/m^3^ based on the Korean National Ambient Air Quality Standards.[^2^](#_ENREF_2)

† Cox proportional hazards model adjusted for clinical variables. Clinical variables were remaining PM-CHA_2_DS_2_-VASc, PM-CHADS_2_, and PM-HATCH components of age, male sex, heart failure, hypertension, diabetes, stroke/transient ischemic attack, vascular disease including previous history of myocardial infarction or peripheral vascular disease, chronic obstructive pulmonary disease, and PM_2.5_.
